# Editorial: The Non-Coding Transcriptome as a New Player in Intercellular Communication

**DOI:** 10.3389/fmolb.2022.858702

**Published:** 2022-02-23

**Authors:** Yvan Devaux, Florence Pinet, David de Gonzalo-Calvo

**Affiliations:** ^1^ Cardiovascular Research Unit, Department of Population Health, Luxembourg Institute of Health, Strassen, Luxembourg; ^2^ Univ. Lille, Inserm, CHU Lille, Institut Pasteur de Lille, U1167 - RID-AGE-Facteurs de Risque et Déterminants Moléculaires des Maladies Liées au Vieillissement, Lille, France; ^3^ Translational Research in Respiratory Medicine, University Hospital Arnau de Vilanova and Santa Maria, IRBLleida, Lleida, Spain; ^4^ CIBER of Respiratory Diseases (CIBERES), Institute of Health Carlos III, Madrid, Spain

**Keywords:** hormone, communication, microRNA, ncRNA, signal

Since the discovery of non-coding RNAs (ncRNAs), this family of regulatory RNA molecules has gained great attention as a potential source of clinical indicators, with the number of publications evaluating the translation of circulating ncRNAs as biological markers growing exponentially in the last decade.

Some recent reports illustrate the usefulness of circulating ncRNAs as innovative tools to support medical-decision making in complex conditions (Blanco-Domínguez et al., 2021; Magen et al., 2021; Mens et al., 2021). Paradoxically, the biology of ncRNAs as endocrine genetic signals is far from being completely understood. Evidence from previous *in vitro* and *in vivo* studies suggests that ncRNAs may function as intercellular mediators (Cheng et al., 2019; Li et al., 2021). Nevertheless, there are still fundamental gaps that need to be addressed to elucidate the precise role of these molecules as hormone-like mediators (Pinilla et al., 2021). Indeed, the information on mechanisms and pathways implicated in the release/binding of ncRNAs in/to the carriers, target tissue specificity, ncRNAs delivery and the induction of biological responses in the recipient cells is limited. The data on the role of ncRNAs in research fields such as cross-kingdom gene regulation is also scarce (Dávalos et al., 2021). Furthermore, the technical challenges in the isolation of ncRNA carriers, the quantification in biofluids or the evaluation of the endocrine effects in target cells are key limiting factors. Additional studies are fundamental to explore the function of circulating ncRNAs as mediators of physiological and pathological responses. Consequently, the aim of this Research Topic was to promote the investigation on this topic in different diseases such as cancer, metabolic syndrome, respiratory and cardiovascular diseases.

First, Fujimoto et al. discussed the pathological roles and clinical usefulness of vascular microRNAs (miRNAs) contained in endothelial cell-derived EVs (EC-EVs) in respiratory diseases, in particular on chronic obstructive pulmonary disease, pulmonary hypertension and acute respiratory distress syndrome. The results presented suggest the crucial role of EC-EVs on lung homeostasis and how the disruption of the secretory profiles in pathological conditions drives the progression of respiratory diseases and their comorbidities. In the same way, Bartsch et al. presented the most important advances on the role of protein-bound and EV-bound ncRNAs as biomarkers of vascular and valvular diseases and their role as intercellular communicators and regulators of disease pathways. Overall, previous data suggest a strict control of ncRNAs selection and packaging into extracellular carriers and ncRNAs uptake to target cells: from ncRNAs sorting, e.g., the presence of nucleotide sequences in RNAs found in EV (Villarroya-Beltri et al., 2013), to their delivery via specific endocytosis or phagocytosis pathways. Current challenges that should be addressed in the field of ncRNA-based therapeutics include the application method and the mode of transportation. In this scenario, Tapparo et al. evaluated a novel technology to vehicle miRNAs through EVs. Co-incubation emerges as a promising alternative method for the EV cargo enrichment maintaining EV integrity and stability. Furthermore, serum EV co-incubation with miR-126 enhanced their pro-angiogenic properties both *in vitro* and *in vivo* by increasing the capacity to induce capillary-like structure formation of human EC. These findings were not observed when serum EV were loaded with anti-angiogenic miRNAs, i.e., miR-19b, which supports the selective biological effect mediated by the carried miRNA.

Li et al. explored the role of long non-coding RNAs (lncRNAs) as competing endogenous RNAs (ceRNAs) in metabolic syndrome by analyzing molecular networks in circulating EVs isolated from whole blood. The results showed that metabolic syndrome alters the cargo of circulating EVs and the lncRNA-associated ceRNA network. Importantly, the authors suggested that the ceRNA network within the EVs may play a role in the development and complication of the syndrome, including cancer. In this sense, Wang et al. conducted a comprehensive review of studies on ncRNAs, including miRNAs, lncRNAs and circular RNAs (circRNAs), as regulators of death associated protein kinases (DAPKs) family. Using bioinformatic analyses, the authors described regulatory networks linking ncRNAs and DAPKs which ultimately could be crucial to understand malignant tumor development. Supporting the role of ncRNAs in cancer, Smith et al. reviewed ncRNA molecules that directly or indirectly affect Frizzled protein expression and signaling, receptors implicated in cancer progression. Targeting ncRNAs constitutes an additional approach to inhibiting oncogenic pathways or enhancing tumor-suppressive pathways. Of note, the use of nanoparticles represents an interesting strategy to deliver ncRNA drugs due to the increased stability, circulation time and target accuracy compared to other vectors.

In summary, although previous evidence suggest the potential role of circulating ncRNAs as endocrine genetic signals, further research is still needed to provide a comprehensive overview of the non-coding transcriptome as a determining factor in long-distance signaling. These advances will provide a better understanding of different pathologic conditions, new therapeutic strategies and additional information on biomarkers with clinical application.
